# Formation and hydration of eco-friendly cement using industrial wastes as raw materials

**DOI:** 10.1038/s41598-021-94148-x

**Published:** 2021-07-20

**Authors:** K. Baltakys, T. Dambrauskas, D. Rubinaite, R. Siauciunas, A. Grineviciene

**Affiliations:** Department of Silicate Technology, Kaunas University of Technology, Radvilenu 19, 50254 Kaunas, Lithuania

**Keywords:** Inorganic chemistry, Materials chemistry

## Abstract

In this work, the optimal conditions of the synthesis of eco-friendly cement by using industrial wastes as well as the peculiarities of its early stage hydration were investigated. The eco-friendly cement was synthesized within the 1000–1250 °C temperature range when the targeted composition was 60% of belite, 20% of ye’elimite, and 20% of brownmillerite. It was determined that the optimal sintering temperature for eco-friendly cement is 1100 °C because the primary compounds were fully reacted, and hydraulic active compounds were dominant in the products. Microcalorimetry analysis was performed for the investigation of early stage hydration. The best results of hydration were obtained with the eco-friendly cement which was produced by using mixtures with silica gel waste: three exothermic reactions were observed in the heat evolution curve, while the cumulative heat was equal to 264 J/g after 72 h. Additionally, the sequence of compounds formation during the first day of hydration was analyzed. It was determined that the composition of the initial mixture impacts the hydration rate of synthetic eco-friendly cement; however, it did not affect the mineralogical composition of the hydration products. These results were confirmed by XRD, STA, and SEM analysis.

The increasing needs of the society in the light of the growing economy and industrial development causes intensive consumption of natural resources and generation of even more waste. According to Eurostat data^1^, in 2018, the total waste generated in the European Union amounted to more than 2.3 billion metric tons, of which, about 180 million tons (10.6%) was represented by waste stemming from manufacturing activities. For these reasons, the management of industrial waste and by-products is a growing field of research, which requires to define the optimization of sustainable manufacturing and waste generation^2^.

One of the types of solid industrial waste, specifically, granite cutting waste (GCW), is produced by extracting and cutting/polishing granite stone into the desired shapes^3,4^. According to the literature^5,6^, about 20–30% of granite global production ends up being a by-product, of which, millions of tons are currently stored in landfill sites. Meanwhile, another toxic waste, AlF_3_-rich silica gel (which contains up to 10% F^-^ ions) is formed by the neutralization of hexafluorosilicic acid with aluminum hydroxide in the course of the manufacturing process of aluminum fluoride. Worldwide fertilizer manufacturers produce about 120,000 tons of this waste per year and discharge it in landfill sites^7^. Thus, in order to reduce environmental pollution and conserve natural resources, it is essential to properly dispose of such by-products.

One way to utilize this waste is to use it in the construction industry, especially in cement production. It is known that the production of the most common ordinary Portland cement (OPC) is imposing a number of problems: 1) high sintering temperature (about 1450 °C), which results in enormous energy consumption (2–3% of the global energy consumption); 2) its production constitutes 5–8% of global CO_2_ emissions; 3) a huge amount of natural resources is consumed^8–14^. Due to such issues, in recent decades, high expectations have been placed on the new generation of environmentally friendly cementitious materials—eco-friendly cement^15^. Scientific works^16–18^ demonstrate that the production of non-Portland binders (e.g., calcium aluminate (CA), calcium sulfoaluminate (CSA), belite-CSA (BCSA), and belite-ye’elimite-ferrite (BYF) binders) leads to a reduction of the carbon footprint. For instance, Hanein et al.^19^ estimated that the production of CSA clinker reduces CO_2_ net emissions by 25–35% relative to OPC, depending on the phase composition of the final CSA clinker. Furthermore, the above mentioned manufacturing of non-Portland binders requires lower energy consumption (the firing temperature is typically ~ 200 °C lower than OPC); in addition, clinker is softer and more friable than Portland cement clinker, and it lowers the energy amount needed for the grinding process^12,20–22^.

Generally, low-CO_2_ clinker is produced from limestone, bauxite, and calcium sulfate; however, the high cost of natural sources poses a major economic challenge for non-Portland binders^23^. For this reason, the application of different waste rich in CaO, SiO_2_, and Fe_2_O_3_ to produce eco-friendly cement is in great demand. According to the literature^24–29^, such wastes as ferroalumina, fly ash, marble sludge waste, phosphogypsum, baghouse dust, ceramic waste, and others can be applied for the manufacturing of CSA, BCSA, and BYF cement. The mineral composition of low-CO_2_ clinkers usually includes belite (β-C_2_S), ye’elimite (C_4_A_3_Ŝ), tetracalcium alumino ferrite (C_4_AF), and, as minor phases, some calcium aluminates (such as CA, C_12_A_7_, and C_3_A)^30,31^. Due to the fast hydration of aluminates and sulfoaluminate, usually, the source of sulphate (gypsum, basanite, or anhydrite) is added to the final clinker thus ensuring the required rate of hydration. During the early stages of hydration, ye’elimite and the sulfate source dissolve, and the main crystalline hydration products—ettringite (AFt phase; C_6_AŜ_3_H_32_) and monosulfate (AFm phase; C_4_AŜH_12_)—are formed together with amorphous aluminum hydroxide (AH_3_)^32,33^. In comparison to ordinary Portland cement, the previously listed eco-cements have also shown many advantages, such as fast setting and hardening, early strength and high strength, low shrinkage, corrosion resistance, etc.^34,35^.

There is a substantial sample of papers which have examined the possibility to use GCW or silica gel as an additive or replacement of OPC cement or sand. According to the scientific literature, the addition of a certain amount of GCW or silica gel to cement has a beneficial impact on the concrete mix, as well as on the physical and mechanical parameters of hardened concrete^36–40^. However, most of these papers focused on partial OPC replacement, but information about the application of GCW and silica gel as a raw material in the production of eco-friendly cements is still scarce. For this reason, the aim of this work is to establish the optimal conditions of the synthesis of an eco-friendly cement by using two types of industrial waste (silica gel and granite cutting waste) and to determine the peculiarities of its early stage hydration.

## Materials and methods

### Raw materials

In this work, the following materials were used:

- Calcium carbonate (CaCO_3_, JSC *Naujasis kalcitas*, Lithuania) which purity ≥ 91.0 wt% of CaCO_3_;
- Calcium sulfate hemihydrate (CaSO_4_·0.5H_2_O, *Knauf*, Germany) which consisted of 22.8 wt% of Ca, 18.47 wt% of S, 1.54 wt% of Si and other substances;
- Iron (III) oxide (Fe_2_O_3_, *Honeywell*, Germany) with purity ≥ 97.0 wt% of Fe_2_O_3_;
- Aluminum hydroxide (Al(OH)_3_, *Honeywell*, Germany) with purity ≥ 99.0 wt% of Al(OH)_3_;
- Calcium sulfate dihydrate (CaSO_4_·2H_2_O, *Lach-Ner*, Poland) which consisted of 27.07 wt% of Ca, 20.64 wt% of S, and other substances.

**Figure 1:**
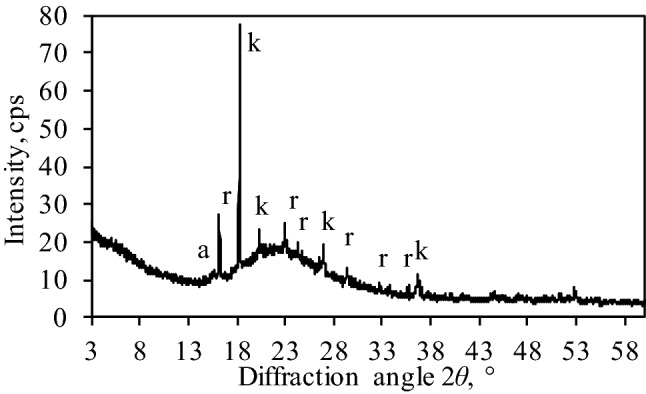
XRD pattern of silica gel waste. Indexes: k-gibbsite; r-aluminum trifluoride trihydrate; a-aluminum hydroxide fluoride hydrate.

Also, two industrial wastes—silica gel waste (JSC *Lifosa*) and granite cutting waste (*Granitas*, Ltd., Lithuania)—were used for the preparation of the initial clinker mixture designs. It was determined that aluminum fluoride production waste—silica gel—consists of gibbsite (Al(OH)_3_, PDF No. 04-011-1369), aluminum trifluoride trihydrate (AlF_3_·3H_2_O, PDF No. 00-035-0627), aluminum hydroxide fluoride hydrate (AlF_1.5_(OH)_1.5_(H_2_O)_0.375_, PDF No. 01-074-0940) and amorphous silicon dioxide (a broad basal reflection within the 18–37° diffraction angle range) (Fig. 1). XRF and chemical analysis revealed that silicon, aluminum and fluorine are the dominant components in the silica gel waste: Si-34.1 wt%, F-8.4 wt%, Al-3.6 wt%.

**Figure 2:**
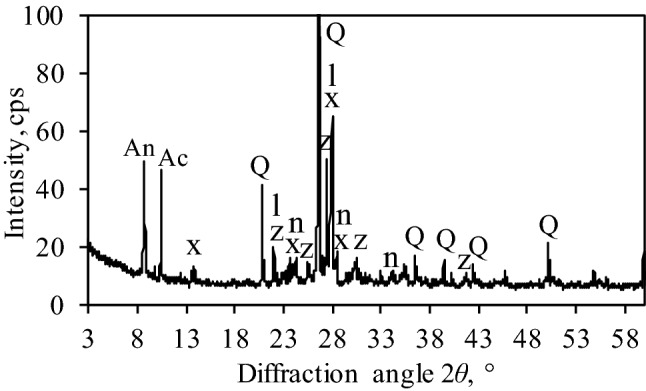
XRD pattern of granite cutting waste. Indexes: An-annite; Ac-actinolite; z-microcline; x-albite; Q-quartz; n-anorthite, l-labradorite.

After examination of the mineral composition of granite cutting waste, the following compounds were identified: quartz (SiO_2_, PDF No. 04-007-0522), microcline (KAlSi_3_O_8_, PDF No. 04-008-1783), annite-1 M (KFe_2_(Si,Al)_4_O_10_(OH)_2_, PDF No. 00-042-1413), anorthite (CaAl_2_Si_2_O_8_, PDF No. 00-041-1486), actinolite (Na_0.08_Ca_1.76_Mn_0.16_Mg_1.88_Fe_2.72_Fe_0.32_Al_0.32_Si_7.68_O_22_(OH)_2_, PDF No. 00-073-2339), albite ((Na,Ca)Al(Si,Al)_3_O_8_, PDF No. 00-041-1480) and labradorite (Ca_0.64_Na_0.35_(Al_1.63_Si_2.37_O_8_), PDF No. 00-083-1371) (Fig. 2). X-ray fluorescence analysis showed that granite cutting waste consists of 27.8 wt% of Si, 7.2 wt% of Fe, 6.9 wt% of Al, 4.0 wt% of K, 3.9 wt% of Ca, 1.24 wt% of Mg, and a minor fraction of other elements, such as Na, Ti, P, Ba and Mn.

### Preparation of eco-friendly cement

Two compositions of eco-friendly cement were prepared by mixing industrial wastes and natural raw materials. The targeted mineralogical composition of eco-friendly cement was selected according to the composition of such a kind of cements presented in the literature data^30,41,42^. The main difference between the compositions of S1 and S2 is that S2 sample was prepared by using natural raw materials and GCW, meanwhile, in S1 sample, a part of GCW was replaced with silica gel by maintaining the same targeted mineralogical composition (Table 1). The values of the experimental compositions were slightly lower than theoretically calculated, but the deviations from the target composition were less than 3%.

**Table 1:** Targeted mineralogical composition of eco-friendly cement, dosages of raw materials and oxide composition of the obtained mixtures (excluding H_2_O and CO_2_).

Sample	S1	S2
**Targeted mineralogical composition**, **wt%**		
2CaO·SiO_2_	60	
4CaO·3Al_2_O_3_·SO_3_	20	
4CaO·3Al_2_O_3_·Fe_2_O_3_	20	
**Raw material**, **wt%**		
Calcium carbonate	66.68	66.60
Gypsum	3.17	3.19
Granite cutting waste	14.35	19.18
Iron (III) oxide	2.50	2.18
Aluminum hydroxide	9.48	8.85
Silica gel waste	3.82	0
**Obtained composition**, **%**		
CaO	55.64	
SiO_2_	20.93	
Al_2_O_3_	14.23	
SO_3_	2.62	
Fe_2_O_3_	6.58	

In order to prepare the initial mixtures, the raw materials were weighed, poured into sealed plastic containers with 2 grinding bodies (so that to ensure homogenization quality) and homogenized for 45 min at 34 rpm by using a homogenizer *TURBULA TYPE T2F*. In order to ensure the required fineness of the reactants, the homogenized starting mixtures were ground for 3 min at 850 rpm in a laboratory vibrating disc mill *Pulverisette 9*.

20 g of the initial mixture was poured into a 36 mm diameter cylinder and compressed by using a hydraulic press (10 Mpa) at 0.5 MPa/s pressing rate. The initial tablets, about 11 mm in height, were sintered in four steps in a high temperature furnace (*Nabertherm HTC 03/16*). Firstly, the temperature was increased to 900 °C by using a 5 °C/min heating rate, and, when 900 °C had been reached, the samples were maintained for 30 min. Afterwards, the temperature was further raised from 900 to 1000 °C at a heating rate of 2 °C/min, and the samples were being maintained at 1000 °C for 1 h. The same procedure was repeated at different temperatures, i.e., the temperature was increased from 900 to 1050 °C, to 1100 °C, to 1150 °C, to 1200 °C, and to 1250 °C. After the synthesis, the obtained samples were simultaneously crushed manually and quickly cooled to room temperature by forced airflow to prevent the formation of γ-C_2_S which does not exhibit hydraulic activity. The room temperature was reached within 5 min. Thee above outlined calcination mode was selected to completely eliminate moisture from the samples and to decompose calcium carbonate (~ 900 °C)^43^.

The obtained eco-friendly cement samples were mixed with 5 wt% of gypsum (45 min; 34 rpm) and ground for 3 min at 850 rpm (S_a_ = 350–400 m^2^/kg). The gypsum additive was used to slow down the initial hydration of cement compounds^34,44^.

### Analytical techniques

The samples were characterized by powder X-ray diffraction (XRD; with a *D8 Advance* X-ray diffractometer), X-ray fluorescence spectroscopy (XRF; with a *Bruker X-ray S8 Tiger WD* spectrometer), simultaneous thermal analysis (STA; with a *Linseis PT1000* instrument), and scanning electron microscopy (SEM; with a *JEOL JSM-7600F* instrument).

XRF was performed on a *Bruker X-ray S8 Tiger WD* (Germany) spectrometer equipped with a Rh tube with the energy of up to 60 keV. Powder samples (passed through a 63 μm sieve and pressed to cylindrical tablets of 5 × 40 mm) were measured in He atmosphere, and the data was analyzed with *SPECTRAPlus QUANT EXPRESS* standardless software.

XRD analysis was performed on a *D8 Advance* diffractometer (*Bruker AXS*, Karlsruhe, Germany) operating at the tube voltage of 40 kV and tube current of 40 mA. The X-ray beam was filtered with Ni 0.02-mm filter to select the CuKα wavelength. Diffraction patterns were recorded in a Bragg–Brentano geometry by using a fast counting detector *Bruker LynxEye* based on the silicon strip technology. The samples were scanned over the range 2*θ* = 3–70° at a scanning speed of 6° min^−1^ while using the coupled two theta/theta scan type.

STA was performed with a *LINSEIS STA PT 1000* (Germany) thermal analyzer. DSK-TGA parameters were as follows: temperature increase rate – 10 °C/min, temperature range – 30–1000 °C, standard empty Pt-10 wt% Rh crucibles, atmosphere in the furnace—nitrogen, the sample mass – 10 mg. The measurement accuracy was ± 3 °C.

SEM was performed by using a *JEOL JSM-7600F* (Japan) instrument at an accelerating voltage of 10 kV, at a working distance of 8.6 mm.

The early-stage hydration of eco-friendly cement samples was investigated by using an eight-channel *TAM Air III* isothermal calorimeter. Glass ampoules (20 ml) each containing 3 g of dry eco-friendly cement were placed in the calorimeter, and the injection units for each ampoule were filled with amounts of water equivalent to a water/solid ratio of 0.5. After a steady temperature of 25 °C had been reached, the water was injected into the ampoules and mixed inside the calorimeter with the dry material for 20 s (at a frequency of 2–3 s^–1^). The heat evolution rate was then measured over a period of 72 h. Repetition of the measurements showed deviations in the total heat below 3% for the samples of a similar type. Apart from the initial minutes of water addition and mixing, the heat evolution rates were essentially identical.

## Results and discussion

### Synthesis of eco-friendly cement

**Figure 3:**
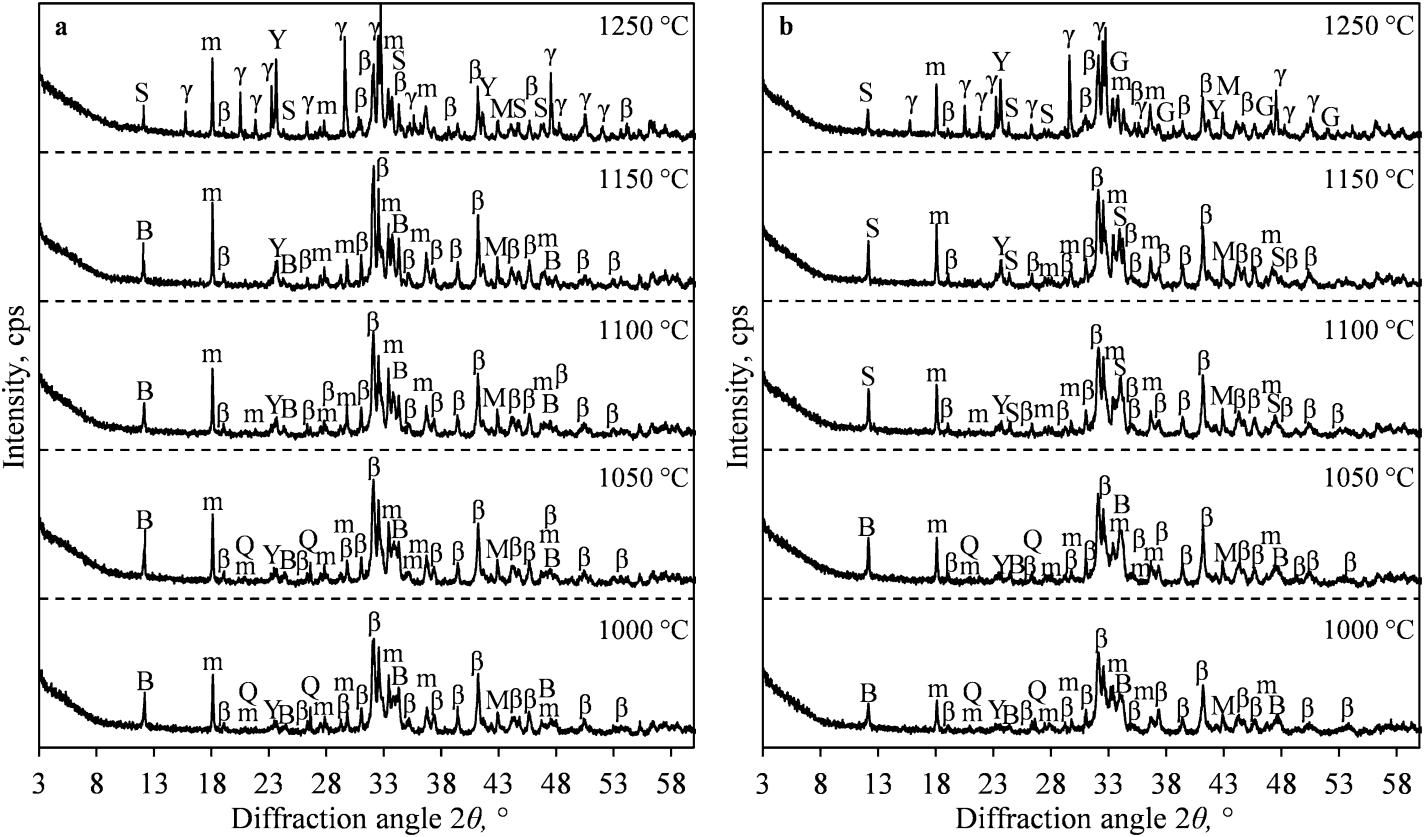
XRD patterns of S1 (**a**) and S2 (**b**) samples sintered at different temperatures. Indexes: Q-quartz; S-srebrodolskite; m-mayenite; β-larnite (β-C_2_S); γ-dicalcium silicate (γ-C_2_S); M-periclase; Y-ye’elimite; B-brownmillerite; G-gehlenite.

In the first stage of this research, the influence of the calcination temperature on the formation of eco-friendly cement was investigated. It was determined that, after solid sintering at 1000 °C and 1050 °C temperatures, quartz did not fully react in both samples because the diffraction peaks characteristic of quartz (PDF No. 00-005-0490) were still observed in XRD patterns (Fig. 3).

It was concluded that the increase in temperature (to 1100 °C) positively affected the interaction degree between raw materials because the main diffraction peaks of quartz were not detected in XRD patterns (Fig. 3). It was determined that, under these conditions of synthesis, larnite (β-C_2_S, β-Ca_2_SiO_4_; PDF No. 00-033-0302), mayenite (Ca_12_Al_14_O_33_, PDF No. 04-014-8824), magnesium oxide (MgO, PDF No. 00-043-1022), brownmillerite (Ca_2_Fe_0.741_Al_1.259_O_5_, PDF No. 04-014-6641) and ye’elimite (Ca_4_Al_6_O_12_SO_4_, PDF No. 00-033-0256) were formed in S1 sample (Fig. 3). It is worth mentioning that the mineral composition of S2 sample slightly differs from that of S1 sample because some of MgO reacted with brownmillerite, and, as a result, srebrodolskite (Ca_2_Mg_0.1_FeAl_0.9_O_5_, PDF No. 04-014-9001) was formed. Further analysis showed that the diffraction peaks of larnite, mayenite, and brownmillerite are more intensive in S1 sample comparing to S2 sample. Probably, the fluorine ions which are present in S1 sample reduce the formation temperature of the latter compounds and increase the orderliness of their crystal structure. According to literature, fluoride ions can intercalate to the structure of cement phases and subsequently form new compounds^45,46^. The ongoing reactions depend on the temperature of sintering, the mineral composition of raw materials, and on the concentration of fluoride ions. Thus, during the clinkering process of S1 sample, fluoride ions could intercalate to the structure of the formed phases, and this way they could form stable fluorine-containing compounds: CaF_2_, calcium-silicate-aluminate, etc. Since the concentration of fluorine in the products is equal to 1%, and the quantity of the formed compounds is low, thus no compounds containing fluorine were identified in the XRD patterns. Also, the identification of such compounds is deteriorated by the intensive diffraction peaks of other cement phases.

The increase in the calcination temperature to 1150 °C had no beneficial impact on the mineral composition and the stability of the formed eco-friendly cement (Fig. 3). However, the temperature increment to 1200–1250 °C positively affected the formation of mayenite and ye’elimite because the intensity of the diffraction peaks characteristic to these compounds increased (Fig. 3). Unfortunately, β-C_2_S formed in this temperature was not stable, and it subsequently recrystallized to another dicalcium silicate – γ-C_2_S (γ-Ca_2_SiO_4_, PDF No. 00-049-1672) during the cooling process of the clinker. It should be noted that the formation of γ-C_2_S in cement is undesirable because of its low hydration activity^47^. According to the literature^17^, in order to avoid β-C_2_S recrystallization to γ-C_2_S, fast cooling or stabilizing agents should be used. However, even fast cooling (room temperature was reached within 5 min) did not prevent the formation of γ-C_2_S. Meanwhile if the additives (stabilizing agents) are used for the production, the cost of the final product increases. Also, under these calcination conditions, brownmillerite recrystallized into srebrodolskite in S1 sample, while a new product of synthesis—gehlenite (Ca_2_Al_2_SiO_7_, PDF No. 00-009-0216)—was formed in S2 sample.

In order to determine the hydration activity of the synthesized eco-friendly cement, the samples calcined at 1100 °C and 1150 °C were analyzed. According to the initial composition and the calcination temperature, the samples calcined at 1100 °C were named S1-1100 and S2-1100, while those calcined at 1150 °C were further referred to as S1-1150 and S2-1150.

### Microcalorimetric study

According to the literature^34,44^, a large number of simultaneous chemical reactions take place during the hydration of different kinds of cement; therefore, microcalorimetry is one of the most accurate methods to monitor the global reaction process on the grounds of the rate of heat production.

**Figure 4:**
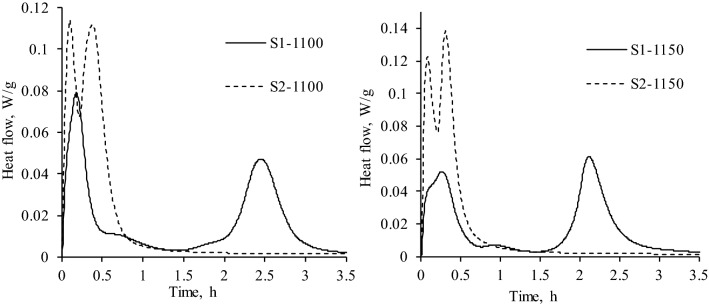
Curves of heat evolution rate of eco-friendly cement samples.

The heat evolution curves of S1 samples showed three exothermic reactions under all experimental conditions (Fig. 4). It was determined that, in S1-1100 and S1-1150 samples, the first hydration reaction lasted for ~ 0.5 and ~ 0.6 h, respectively, while the heat flow maximums (0.07614 W/g for S1-1100 and 0.050 W/g for S1-1150) were reached after 9–13 min. The initial hydration reaction can be ascribed to the heat released during the initial wetting of cement samples as well as to the interaction of aluminate phases (mayenite) with water and gypsum^30^.

**Figure 5:**
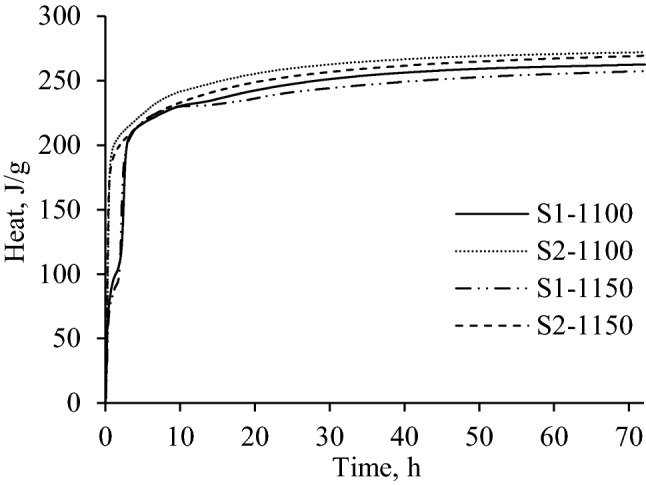
Curves of the cumulative heat of eco-friendly cement samples.

After the initial reaction, the second exothermic reaction which can be related with the further hydration of ye’elimite or the interaction of brownmillerite was observed^48,49^ (Fig. 4). It was determined that it lasted for 1 h, and the heat flow maximum was equal to 0.007–0.01 W/g. The main hydration reaction (3rd exothermic reaction) in S1-1100 sample started after 2 h and ended after 3 h of hydration, while this process was observed ~ 0.5 h earlier in S1-1150. It was determined that the maximum value of the heat flow was 1.3 times lower in S1-1100 sample (0.047 W/g) than in S1-1150 (0.06 W/g). Finally, the microcalorimetry curves showed that, after the main exothermic reaction, a slow hydration period began, which was controlled by the diffusion process (Fig. 4). It was estimated that, during the early-stage hydration, the cumulative heat (72 h) did not depend on the calcination temperature because both samples showed similar values (Fig. 5 and Table 2). It is worth mentioning that the listed values are close to those presented in the relevant literature^42,43^.

**Table 2:** Cumulative heat of eco-friendly cement samples.

Sample	S1-1100	S1-1150	S2-1100	S2-1150
Total heat after 72 h, J/g	264	259	274	270

It was determined that the hydration process was greatly affected by the composition of the initial mixture because S2 samples showed only two hydration reactions (Fig. 4). It was observed that the maximum of the initial reaction in S2 samples was reached after 5 min of hydration (Fig. 4). In addition, the values of the heat flow maximum of S2-1100 (0.114 W/g) and S2-1150 (0.1225 W/g) were about 1.5 and 2.3 times higher in comparison with S1-1150 and S1-1100, respectively (Fig. 4). Furthermore, due to the rapid interaction between eco-friendly cement particles and water, the main hydration reaction immediately followed the initial reaction and ended after 1 h of hydration. It was determined that the values of the total heat of S2 samples after 72 h of hydration were slightly higher than in S1 samples (Table 3), however, it is not recommended to use S2 samples for the manufacturing of cement because the hydration process is too short as it ends after 1 h.

Presumably, the differences between S1 and S2 samples are manifested due to the presence of F^−^ ions in the initial mixture (from the silica gel waste). According to the literature^50,51^, the quantity of F^−^ ions in the primary mixture and the formed compounds containing fluorine during sintering have a crucial effect on the hydration process of cement. In the case of a low quantity of fluoride ions added to the primary mixtures, the hydration process of cement slows down, and, as a result, the setting time is delayed. On the other hand, if the concentration of F^−^ ions is increased in the mixture, these ions accelerate the dissolution of Ca^2+^ ions, which leads to the promotion of dissolution of silicates and a decrease of the setting time/induction period. As the results of other authors showed, the critical concentration of F^−^ (i.e., the concentration which accelerates or slows down hydration) depends on the mineralogical composition of cement, water-cement ratio, and the environment of hydration. By summarizing literature data and the results of the microcalorimetry test, it is reasonable to assume that, during the wetting of S1 sample particles, on the surface of the particles, insoluble fluorine-containing compounds are accumulated, which temporarily inhibits the hydration of the cement, and, as a result, the induction period is prolonged.

### Mineralogical composition of hydration products

It is known that the properties of cement depend on the reactivity of the cement phases and the mineral composition of the hydration products^30^. Therefore, in order to evaluate the formation of hydration products, hydration experiments of S1-1100 and S2-1100 samples were performed at 25 °C temperature in a thermostat. After the selected time had elapsed, hydration was halted by using ethanol. Later on, these samples were crushed to powder, dried at 50 ± 5 °C temperature and put through a sieve (80-μm mesh). The selected durations of the stopped reactions coincide with the duration intervals of exothermic reactions.

**Figure 6:**
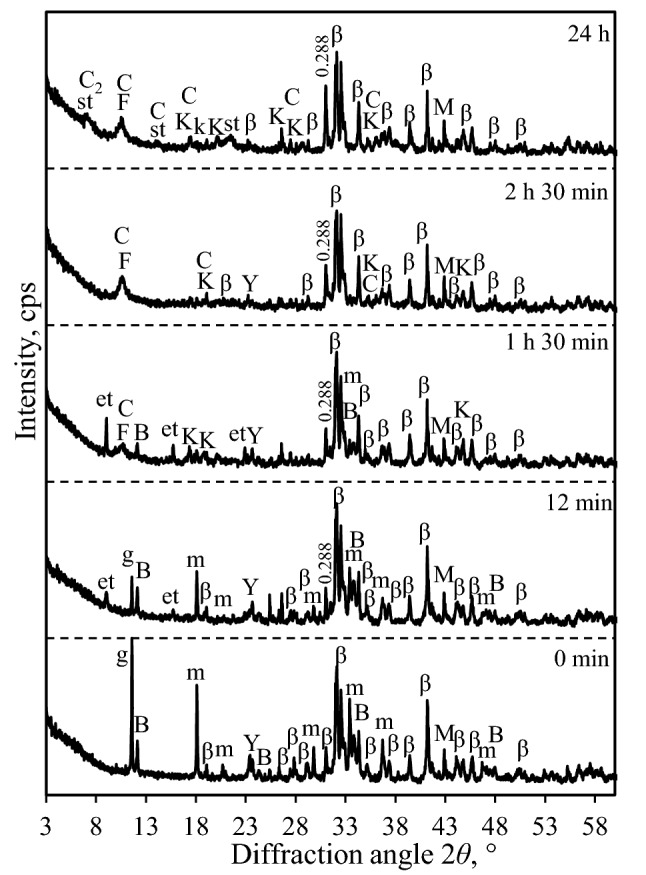
XRD patterns of S1-1100 sample after different duration times of hydration. Indexes: m-mayenite, β-larnite (β-C_2_S), M-magnesium oxide, Y-ye’elimite, B-brownmillerite, g-gypsum, et-ettringite, st-stratlingite, k-gibbsite, K-katoite, C-CAH_10,_ C_2_-C_2_AH_8_, F-ferro-actinolite.

It was determined that, at the beginning of hydration of S1-1100 sample (12 min), intensive interaction between gypsum mayenite (C_12_A_7_), (CŜH_2_) and water (H) was taking place, and it resulted in the formation of ettringite (C_6_AŜ_3_H_32_) (Fig. 6). It was noted that the main diffraction maximums of CŜH_2_ and C_12_A_7_ decreased by about 74% and 48%, respectively (Table 3). According to the results of XRD and those presented in the literature^52,53^, the formation of ettringite can be described by the following reaction:

1 $$ {\text{C}}_{12} {\text{A}}_{7} + \, 12{{\text{C}\hat{\text{S}}\text{H}}}_{2} + \, 113{\text{H }} \to \, 4{\text{C}}_{6} {{\text{A}\hat{\text{S}}}}_{3} {\text{H}}_{32} + \, 3{\text{AH}}_{3} $$

**Table 3:** Changes of the main diffraction peaks characteristic to the synthesis products of S1-1100 sample.

Compound	Time (h)					
	Intensity, cps					
	0 min	12 min	1 h 30 min	2 h 30 min	3 h 30 min	24 h
Larnite (0.279 nm)	943	940	935	866	854	762
Mayenite (0.489 nm)	780	409	85	–	–	–
Gypsum (0.763 nm)	1174	297	–	–	–	–
Brownmillerite (0.723 nm)	235	220	102	–	–	–
Ye’elimite (0.376 nm)	205	159	114	–	–	–

The beginning of ye’elimite (C_4_A_3_Ŝ) hydration was also observed: the main diffraction peak typical to this compound decreased by about 22% (Fig. 6, Table 3). During this process, a number of simultaneous reactions can proceed (Eqs. 2–4), which may lead to the formation of ettringite and monosulphate (C_4_AŜH_12_) in the obtained products (Fig. 6)^54,55^:

2 $$ {\text{C}}_{4} {\text{A}}_{3} {\hat{\text{S}}} + \, 18{\text{H }} \to {\text{C}}_{4} {{\text{A}\hat{\text{S}}\text{H}}}_{12} + \, 2{\text{AH}}_{3} $$

3 $$ 2{\text{C}}_{4} {\text{A}}_{3} {\hat{\text{S}}} + \, 2{{\text{C}\hat{\text{S}}\text{H}}}_{2} + \, 52{\text{H }} \to {\text{ C}}_{6} {{\text{A}\hat{\text{S}}}}_{3} {\text{H}}_{32} + {\text{ C}}_{4} {{\text{A}\hat{\text{S}}\text{H}}}_{12} + \, 4{\text{AH}}_{3} $$

4 $$ {\text{C}}_{4} {\text{A}}_{3} {\hat{\text{S}}} + \, 2{{\text{C}\hat{\text{S}}\text{H}}}_{2} + \, 36{\text{H }} \to {\text{ C}}_{6} {{\text{A}\hat{\text{S}}}}_{3} {\text{H}}_{32} + \, 2{\text{AH}}_{3} $$

According to Eqs. (1–4), aluminum hydroxide (AH_3_) should crystallize into products, however, the latter compound is amorphous and cannot be detected in XRD patterns (Fig. 6). It was observed that belite (C_2_S) and brownmillerite (C_4_AF) remained stable under these conditions of hydration.

**Figure 7:**
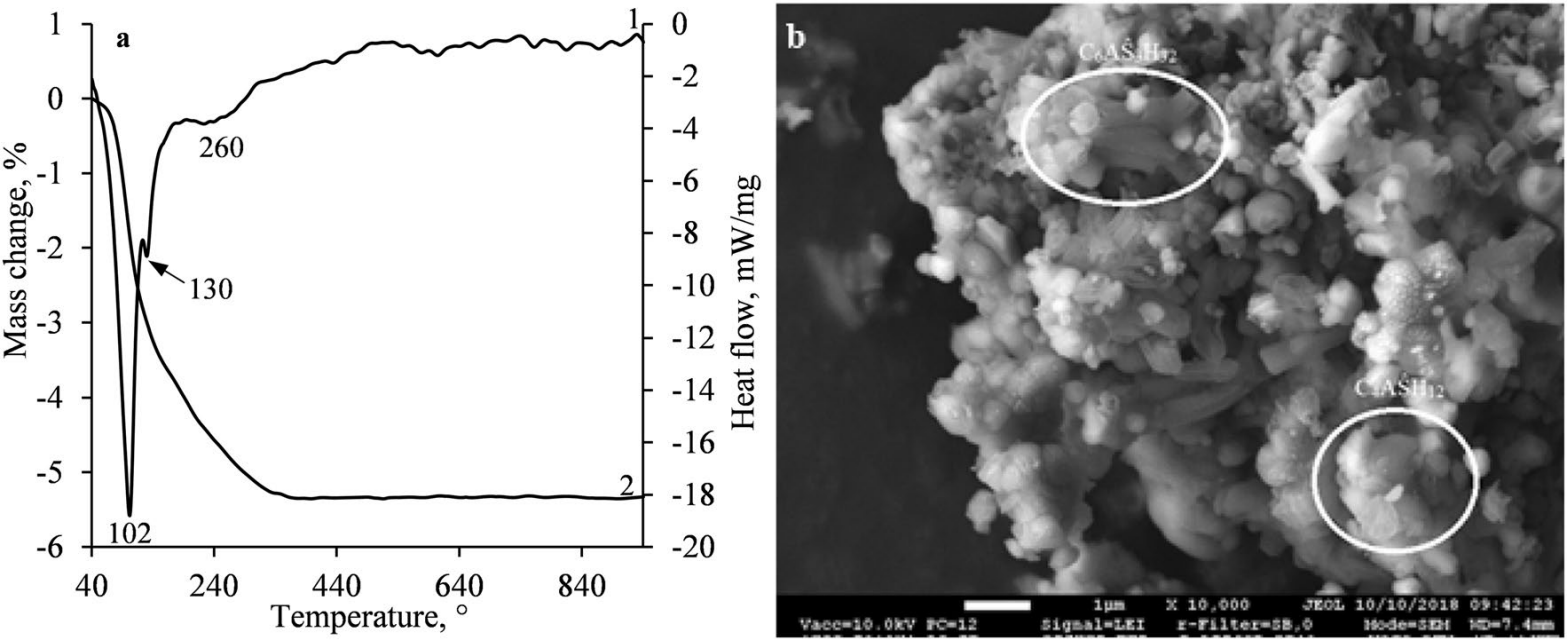
STA (**a**) curves (1-DSC; 2-TGA) and SEM micrographs (**b**) of S1-1100 sample after 12 min of hydration.

The obtained results were confirmed by the data of STA. The first endothermic effect at ~ 102 °C temperature reflects the removal of adsorption water and the dehydration of compounds of an amorphous structure^54,56^ (Fig. 7a). Meanwhile, the shoulders at 130 °C temperatures can be assigned to the decomposition of ettringite and monosulphate^54,57^. It is notable that the results of STA confirmed the formation of aluminum hydroxide during reactions (1–4) because the endothermic effect at 260 °C corresponding to the decomposition of AH_3_ was observed (Fig. 7a). The formation of ettringite and monosulphate was also confirmed by SEM analysis (Fig. 7b): the crystals of ettringite and plate-like crystals of monosulphate were observed. Additionally, small, rounded particles of larnite were detected.

It was determined that, by prolonging the duration of hydration to 1 h 30 min, i.e., after the second exothermic reaction (Fig. 4a), the intensity of the diffraction peaks of mayenite and ye’elimite decreased by about 90% and 50%, while those of ettringite increased by 3 times (from 96 to 288 cps) (Table 3, Fig. 6). As expected, gypsum fully reacted because the diffraction peaks characteristic to this compound were not detected in XRD patterns. It is worth noting that, due to the specificity of the C_4_AŜH_12_ structure, some SO_4_^2–^ groups can be replaced by other anions (Cl^−^, NO_3_^−^, CO_3_^2−^, etc.); hence, Kuzel’s salt (C_4_AŜ_0.5_Cl_0.5_H_11_) and carbonated monsulphate (C_4_AŜ_0.5_(CO_2_)_n_H_x_, C_4_AĈ_0,5_H_x_ and C_4_AĈH_x_) can potentially be identified in the hydration products. Furthermore, the hydration of brownmillerite (C_4_AF) started under these hydration conditions, which led to the formation of various compounds in the products: hydrogarnet (C_3_AFSH_4_), katoite (C_3_AH_6_), portlandite (CH), calcium ferrite hydrate (CFH), ferro-actinolite (C_2_FS_8_H_2_) (Ca_2_Fe_5_Si_8_O_22_(OH)_2_, PDF No. 00-023-0118), etc. However, the quantity of some presently mentioned formed compounds was small, thus the compounds cannot be clearly distinguished in the XRD patterns. The formation of new compounds can be described by the following reactions^54,58,59^:

5 $$ {\text{C}}_{{4}} {\text{AF}} + {\text{ 7H}} \to {\text{C}}_{{3}} {\text{AH}}_{{6}} + {\text{CFH}} $$

6 $$ {\text{C}}_{{2}} {\text{S }} + {\text{ C}}_{{4}} {\text{AF }} + {\text{ 7H}} \to {\text{C}}_{{3}} {\text{AFSH}}_{{4}} + {\text{ 3CH}} $$

7 $$ {\text{C}}_{4} {\text{AF }} + \, 3{{\text{C}\hat{\text{S}}\text{H}}}_{2} + \, 27{\text{H }} \to \, C_{6} {{\text{A}\hat{\text{S}}}}_{3} {\text{H}}_{32} + {\text{ CFH}} $$

8 $$ {\text{C}}_{{4}} {\text{AF }} + { 1}0{\text{H }} + {\text{ 2CH}} \to {\text{C}}_{{6}} {\text{AFH}}_{{{12}}} $$

It was determined that, after 2.5 h of hydration of S1-1100 sample, mayenite, brownmillerite and ye’elimite fully recrystallized into the hydration products (Fig. 6, Table 3). Meanwhile, two calcium aluminate silicates—katoite (C_3_AH_6_, PDF No. 04–017–1504) and metastable phase (CAH_10_)—were also observed in the products (Fig. 6). The presently mentioned compounds mainly formed during the hydration of mayenite and, partially, during the hydration of ye’elimite and brownmillerite^31,57^. It was determined that about 19% of larnite was hydrated, and stratlingite (C_2_ASH_8_, PDF No. 00-046-1348) with semi-crystalline calcium silicate hydrates was formed after 24 h of hydration:

9 $$ {\text{C}}_{{2}} {\text{S }} + {\text{ AH}}_{{3}} + {\text{ 5H}} \to {\text{C}}_{{2}} {\text{ASH}}_{{8}} $$

Moreover, a new hydration product—metastable phase C_2_AH_8_—was identified under these hydration conditions. It is worth highlighting that, even after 24 h of hydration, magnesium oxide did not show any hydration activity (Figs. 3a, 6).

**Figure 8:**
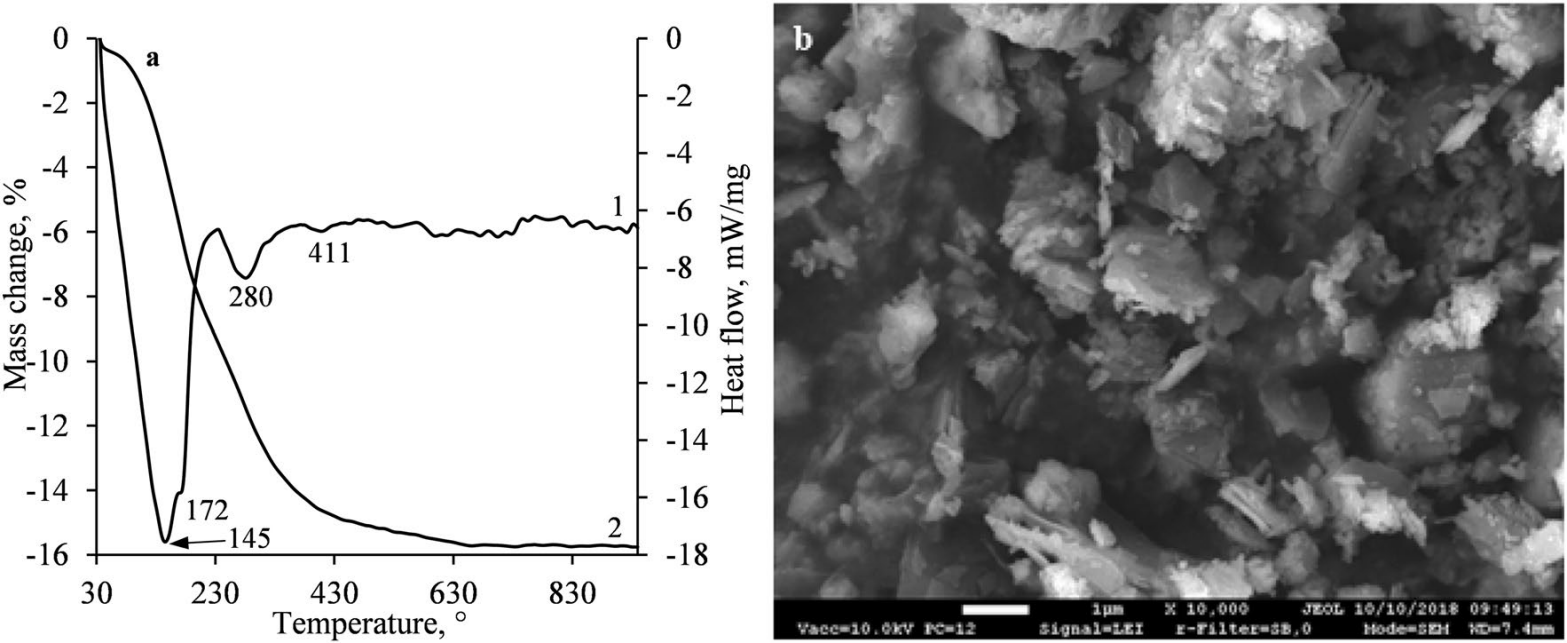
STA (**a**) curves (1-DSC; 2-TGA) and SEM micrographs (**b**) of S1-1100 sample after 24 h of hydration.

The previously obtained results were verified by STA and SEM analysis. In the DSC curve, the heat of the first endothermic effect increased several times (Figs. 7a, 8a), thus it can be stated that a large number of hydration products (C_6_AŜ_3_H_32,_ C_4_AŜH_12_, CAH_10_, C_2_AH_8_, CSH, etc.) were formed. Meanwhile, within a broader temperature interval (250–400 °C), the decomposition of hydrogarnet, katoite and aluminum hydroxide was observed^31,54,57,60^. However, the decomposition of the presently mentioned compounds was prominently overlapped and could not be compared (Fig. 8a). The small endothermic effect at 411 °C can be attributed to the decomposition of portlandite. It is worth mentioning that, after 24 h of hydration, the loss on ignition of S1-1100 sample increased by about 3 times, i.e., from 5.3% (after 12 min) to 15.4% (after 24 h) in comparison with the sample which was obtained after 12 min of hydration. The SEM micrograph of S1-1100 sample showed particles of an irregular shape together with plate-like crystals and fragments of unreacted larnite (Fig. 8b).

**Figure 9:**
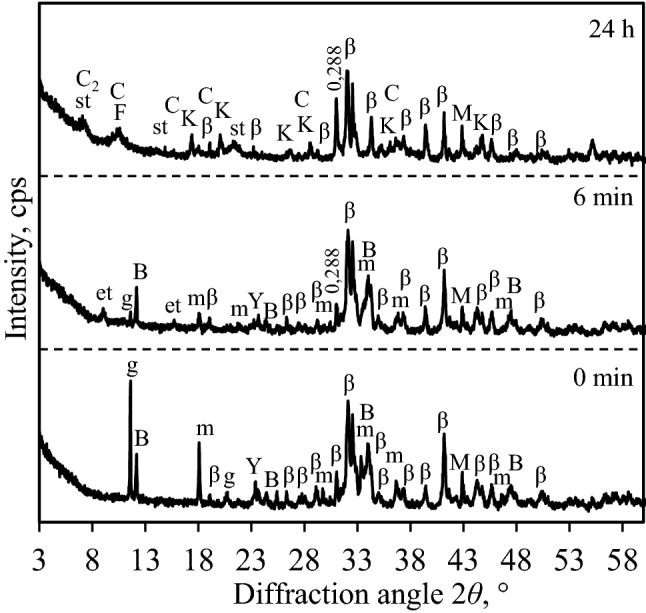
XRD patterns of S2-1100 sample after different durations of hydration. Indexes: m-mayenite, β-larnite (β-C_2_S), M-magnesium oxide, Y-ye’elimite, B-brownmillerite, g-gypsum, et-ettringite, st-stratlingite, k-gibbsite, K-katoite, C-CAH_10,_ C_2_-C_2_AH_8_, F-ferro-actinolite.

As expected, the hydration of the synthesis products and the formation of new compounds in S2-1100 sample proceeded earlier in comparison with S1-1100 (Figs. 6 and 9, Tables 3 and 4). The results of XRD analysis showed that, after 6 min of hydration (i.e., the first exothermic reaction (Fig. 4)), the intensities of the main diffraction peaks corresponding to gypsum and mayenite decreased by more than 90% and 77%, respectively (Table 4, Fig. 9). In addition, the intensity of the brownmillerite and ye’elimite peaks decreased by 35% and 18%, respectively, while, in S1-1100 sample, brownmillerite was stable after the first hydration reaction. It was determined that, within 1 h of hydration, gypsum, mayenite, brownmillerite and ye’elimite had fully reacted (Fig. 9, Table 4). The results of XRD analysis were in good agreement with the data of microcalorimetric analysis because, after 1 h of hydration, the main hydration reaction had finished (Fig. 4). Unfortunately, diffraction peak *d* – 0.288 nm—detected in S1 and S2 samples of the hydration products could not be assigned to the compound in the PDF-4 database which would increase with the aging time (Figs. 6 and 9). It was observed that the hydration of larnite is similar in both samples because, after 24 h, the intensity of the diffraction maximums of this compound decreased by about 17% in S2-1100 sample and by 19% in S1-1000 sample (Tables 3 and 4). It is worth highlighting that the initial mixture composition did not affect the mineralogy of the hydration products but still exerted a significant influence on the hydration process.

**Table 4:** Changes of the main diffraction peaks characteristic to the synthesis products of S2-1100 sample.

Compound	Time (h)						
	Intensity, cps						
	0 min	6 min	12 min	24 min	1 h	3 h 30 min	24 h
Larnite (0.279 nm)	774	755	720	707	681	650	649
Mayenite (0.489 nm)	467	118	106	67	–	–	–
Gypsum (0.763 nm)	870	92	–	–	–	–	–
Brownmillerite (0.723 nm)	350	298	228	97	–	–	–
Ye’elimite (0.376 nm)	120	108	98	89	–	–	–

## Conclusions

It was determined that silica gel and granite cutting wastes can be used as raw materials for the production of eco-friendly cements. The optimal calcination temperature of S1 and S2 samples is 1100 °C because, at a lower temperature, raw materials do not fully react, while, due to a higher temperature (more than 1200 °C), larnite (β-C_2_S) becomes metastable, and, during the cooling process, it recrystallizes into γ-C_2_S which does not exhibit hydration activity. It was determined that the presence of fluoride ions in the initial mixture did not affect the main mineralogical composition of eco-friendly cement, and that the fluoride ions probably intercalated into the structure of the synthesis products or formed a small amount of compounds containing fluorine.

It was determined that the hydration process of synthesized eco-friendly cement depends on the composition of the primary mixture since a significant difference was observed in the microcalorimetry curves of S1 and S2 samples. It can be inferred that the role of F^−^ ions in the hydration of S1 sample is to retard the hydration of cement phases at the initial stages of the hydration process, which delays the setting time of the synthesized cement. Therefore, the main hydration reactions in S1 samples were observed ~ 1.5–2 h later in comparison with S2 samples. Despite the difference of the hydration rate of eco-friendly cement samples, the total amount of the released heat after 72 h of hydration was fairly similar and equal to 260–274 J/g.

The XRD and TG results showed that the hydration of the main cement phases and the formation of the hydration products was significantly delayed in S1 samples in comparison with S2 samples. It was determined that, within 1 h of hydration of S2-1100 sample, C_4_A_3_Ŝ, C_12_A_7_, C_4_AF were fully recrystallized into the hydration products (ettringite, stratlingite, gibbsite, katoite, CAH_10_, C_2_AH_8_, ferro-actinolite). Meanwhile, the above mentioned cement phases fully reacted only after 2.5 h during hydration of S1-1100 sample. Nevertheless, the composition of the primary mixture did not impact the mineralogy composition of eco-friendly cement hydration products obtained after 24 h of hydration.

This paper demonstrates the potential application of GCW and silica gel waste as raw materials in the production of eco-friendly cements. However, further studies are still required to fully determine the physico-chemical and mechanical properties of synthesized eco-friendly cement. Thus, direct continuation of this study would be related to the investigation of curing in an aqueous environment as well as under hydrothermal conditions of the obtained eco-friendly cement.
